# Genetic Association Study with Metabolic Syndrome and Metabolic-Related Traits in a Cross-Sectional Sample and a 10-Year Longitudinal Sample of Chinese Elderly Population

**DOI:** 10.1371/journal.pone.0100548

**Published:** 2014-06-24

**Authors:** Jinghui Yang, Jianwei Liu, Jing Liu, Wenyuan Li, Xiaoying Li, Yao He, Ling Ye

**Affiliations:** 1 Institute of Geriatrics, the General Hospital of the People's Liberation Army, Beijing, China; 2 Beijing Key Lab of Aging and Geriatrics, the General Hospital of the People's Liberation Army, Beijing, China; 3 Department of Epidemiology, Harvard School of Public Health, Boston, Massachusetts, United States of America; 4 Department of Geriatric Cardiology, the General Hospital of the People's Liberation Army, Beijing, China; University of Granada - Q1818002F, Spain

## Abstract

**Background:**

The metabolic syndrome (MetS) has been known as partly heritable, while the number of genetic studies on MetS and metabolic-related traits among Chinese elderly was limited.

**Methods:**

A cross-sectional analysis was performed among 2 014 aged participants from September 2009 to June 2010 in Beijing, China. An additional longitudinal study was carried out among the same study population from 2001 to 2010. Biochemical profile and anthropometric parameters of all the participants were measured. The associations of 23 SNPs located within 17 candidate genes *(MTHFR, PPARγ, LPL, INSIG, TCF7L2, FTO, KCNJ11, JAZF1, CDKN2A/B, ADIPOQ, WFS1, CDKAL1, IGF2BP2, KCNQ1, MTNR1B, IRS1, ACE*) with overweight and obesity, diabetes, metabolic phenotypes, and MetS were examined in both studies.

**Results:**

In this Chinese elderly population, prevalence of overweight, central obesity, diabetes, dyslipidemia, hypertension, and MetS were 48.3%, 71.0%, 32.4%, 75.7%, 68.3% and 54.5%, respectively. In the cross-sectional analyses, no SNP was found to be associated with MetS. Genotype TT of SNP rs4402960 within the gene *IGF2BP2* was associated with overweight (odds ratio (OR)  = 0.479, 95% confidence interval (CI): 0.316-0.724, *p* = 0.001) and genotype CA of SNP rs1801131 within the gene *MTHFR* was associated with hypertension (OR = 1.560, 95% CI: 1.194–2.240, *p* = 0.001). However, these associations were not observed in the longitudinal analyses.

**Conclusions:**

The associations of SNP rs4402960 with overweight as well as the association of SNP rs1801131 with hypertension were found to be statistically significant. No SNP was identified to be associated with MetS in our study with statistical significance.

## Introduction

The metabolic syndrome (MetS) is recognized as a complex disorder of central obesity, high blood pressure, high plasma glucose, and dyslipidemia [1]. People affected by MetS are at an increased risk of severe health complications, such as type 2 diabetes mellitus (T2DM) and cardiovascular disease (CVD) [2],[3]. Heritability estimates for MetS range from 10% to 30% [4]–[6], indicating that MetS is partly heritable. Knowledge of exact genetic factors underlying MetS development may help to explain why the traits of MetS frequently coexist within individuals.

Single-nucleotide polymorphisms (SNPs) are widely present in human DNA sequences and some are considered to be disease-related. In recent years, genome-wide association studies (GWAS) have identified numerous candidate SNPs that are unequivocally associated with metabolic-related traits, including obesity, diabetes, hypertension, and dyslipidemia. However, most of these SNPs were identified and studied in young-age populations, and the number of previous genetic studies in aged participants was limited. Aged participants are expected to have more cumulative environmental exposure and thus be more likely to be affected by gene-environment interaction than younger people. Whether the genetic effects of metabolic-related traits observed in younger groups can be replicated in elders has not be well studied.

In order to detect genes underlying the development of metabolic-related traits, we cross-sectionally analyzed 23 SNPs located in 17 genes among a Chinese elderly population in Beijing. These SNPs include: 1) methylenetetrahydrofolate reductase (*MTHFR*); 2) peroxisome proliferator-activated receptor gamma (*PPARγ*); 3) insulin induced gene 2 (*INSIG2*); 4) transcription factor 7-like 2 (*TCF7L2*); 5) fat mass and obesity associated (*FTO*); 6) potassium inwardly-rectifying channel, subfamily J, member 11 (*KCNJ11*); 7) JAZF zinc finger 1 (*JAZF1*); 8) cyclin-dependent kinase inhibitor 2A (*CDKN2A*); 9) adiponectin, C1Q, and collagen domain containing (*ADIPOQ*); 10) Wolfram syndrome 1 (*WFS1*); 11) CDK5 regulatory subunit associated protein 1-like 1 (*CDKAL1*); 12) insulin-like growth factor 2 mRNA binding protein 2 (*IGF2BP2*); 13) potassium voltage-gated channel, KQT-like subfamily, member 1 (*KCNQ1*); 14) melatonin receptor 1B (*MTNR1B*); 15) insulin receptor substrate 1 (*IRS1*); 16) angiotensin I converting enzyme (*ACE*); and 17) lipoprotein lipase (*LPL*). We further extracted data of 742 participants from the same population to investigate whether individuals that were persistently affected by a particular condition were under a strong influence from markers of genetic susceptibility, and whether individuals that were persistently unaffected by that condition were influenced by the protective effects of certain genes. This subpopulation had completed records from 2001 to 2010, and allowed us to conduct a longitudinal analysis to test our hypotheses.

Analyses were carried out in two stages: i) a cross-sectional study on selected metabolic-related traits and MetS, and ii) a 10-year longitudinal study on persistent metabolic-related traits and MetS.

## Materials And Methods

### Study Participants And Phenotype Definitions

#### Subjects And Study Design

Study participants were from Wanshoulu Community in the Haidian District, Beijing, China. This area represents the local geographic and economic characteristics. Sampling and research methods were described elsewhere [7]. From 2009 to 2010, a total of 2 162 participants were randomly selected based on a 2-stage stratified sampling approach. After excluding subjects with incomplete data and those with age <60 years old, 2 014 (93.2%) subjects were included in the cross-sectional analyses. Furthermore, 742 of these 2 014participants (35%) had completed records back to 2001, which allowed us to conduct a longitudinal study. Subjects in the longitudinal study were grouped based on persistency with metabolic-related traits.

#### Data Collection

Potential participants were invited to a community clinic by letters or telephone calls, and were interviewed with structured questionnaires that covered education level, leisure time physical activity, smoking status, alcohol consumption, family disease history, and individual disease history.

#### Physical Examination

Weight, height, waist circumference (WC), hip circumference, and resting blood pressure were measured according to a standard protocol. Weight was measured in kilograms (with heavy clothes removed and 1 kg deducted from measured weight to account for remaining garments), height was measured in meters (without shoes), and WC was measured in a standing position with a soft tape midway between the lowest rib and the iliac crest. Two measures of resting blood pressure were obtained from the right arm in a sitting position after 30 minutes of rest, with a 5 minutes interval, and averaged. Plasma levels of lipids and glucose were measured using overnight fasting blood specimens. Fasting plasma glucose (FPG) was measured using a modified hexokinase enzymatic method. Concentrations of total cholesterol (TC), high-density lipoprotein cholesterol (HDL-c), low-density lipoprotein cholesterol (LDL-c), and triglycerides (TG) were assessed enzymatically with commercially available reagents following the manufacturer's protocol.

All physical examinations and interviews were carried out by trained nurses and physicians.

#### Definitions Of Overweight And Metabolic-Related Traits

BMI was calculated as body weight in kilograms divided by the square of height in meters. The World Health Organization's definitions of overweight (BMI ≥25 kg/m^2^) and obesity (BMI ≥30 kg/m^2^) and central obesity (waist circumference ≥90 cm in men and ≥80 cm in women) in Asians were used [8],[9]. Dyslipidemia was defined as TC ≥5.8 mmol/L (220 mg/dL), or TG ≥1.7 mmol/L (150 mg/dL), or HDL-c <1.0 mmol/L (40 mg/dL) in men (<1.3 mmol/L (50 mg/dL) in women), or under treatment of dyslipidemia. T2DM was defined by history or 1999 World Health Organization's criteria [10]: FPG concentrations ≥7.0 mmol/L (126 mg/dL) or OGTT (2h) ≥11.1 mmol/L. Hypertension was defined as systolic blood pressure (SBP) ≥140 mmHg, or diastolic blood pressure (DBP) ≥90 mmHg, or by history.

#### Definition Of Mets

MetS status was defined according to the modified International Diabetes Federation (IDF) definition [8]. To have MetS, participants must have central obesity and with any 2 of the following 4 additional factors: 1) high blood pressure (SBP ≥130 mmHg, or DBP ≥85 mmHg, or under treatment); 2) hypertriglyceridemia (TG ≥1.7 mmol/L); 3) low HDL-c (HDL-c <1.0 mmol/L in men or <1.3 mmol/L in women); and 4) hyperglycemia (FPG ≥5.6 mmol/L).

#### Ethics Statemen

The study was approved by the Committee for Medical Ethics of the Chinese PLA General Hospital, and signed informed consents were obtained from all participants.

#### Genotyping

DNA was extracted from the whole peripheral blood sample by the standard proteinase K-phenol-chloroform method. The laboratory staffs were blinded to the identities of the subjects and their MetS status.

The candidate SNPs were selected based on GWAS studies published before August 2010. SNPs identified among Asian populations were considered first [11]–[26]. A total of 23 SNPs from 17 genes that have been confirmed by most of the above studies with minor allele frequency (MAF) >0.1 were chosen for our analyses. The information for reference sequences of the SNPs were searched from the dpSNP database on the National Center for Biotechnology Information (NCBI) website (http://www.ncbi.nlm.nih.gov/snp/).

Genotyping of candidate SNPs was performed using the MassARRAY system. After polymerase chain reaction (PCR) amplification, primer extension products were analyzed by chip-based Matrix-Assisted Laser Desorption/Ionization Time of Flight Mass Spectrometry (MALDI-TOF MS). Extension primers were designed to extend beyond the SNP site by one or two bases using the software Assay Design 3.1. Primer extension as well as PCR were performed according to manufacturer's standard protocols. All PCR amplification procedures were carried out with PCR system. After desalting, approximately 10 nl reaction product were loaded onto a 384-well SpectroCHIP that has been preloaded with patches of crystalline matrix. As quality control measures, four negative controls (water without DNA) were included to detect contamination in each SpectroCHIP. The SpectroCHIPs were analyzed in the fully automated mode with the MALDI-TOF MassARRAY system. SpectroTYPER 4.0 software performs genotype calling automatically using a set of digital filters optimized for mass spectra of oligonucleotides.

The average success rate of genotyping for each polymorphism was 99.5%.

#### Statistical Analysis

Continuous variables were expressed as mean ± standard deviation (SD). Characteristics of study participants were compared using unpaired Student's t-test or Pearson's Chi-square test (or Fisher's exact test) for continuous variables and categorical variables, respectively. Tests of Hardy-Weinberg equilibrium (HWE) and linkage disequilibrium (LD) were performed with HAPLOVIEW software version 4.2 (Document3http://www.broadinstitute.org/haploview). We used multivariable logistic regression models to analyze the associations of candidate SNPs genotypes with MetS and metablic-related traits. Odds ratios (ORs) and 95% confidence intervals (CIs) were reported.

All statistical analyses were performed using SPSS version 16.0 statistic package for windows. All statistical tests were based on 2-sided test, and a *p*-value of <0.05 was considered to be statistically significant. Multiple testing was adjusted using the Bonferroni method when necessary, and a *p*-value of <0.003 was considered as statistically significant.

## Results

### Characteristics

We included 2,014 subjects in the cross-sectional study and 742 subjects in the longitudinal study. The prevalence of overweight, central obesity, T2DM, dyslipidemia, hypertension, and MetS in the cross-sectional study were 48.3%, 71.0%, 32.4%, 75.7%, 68.3% and 54.5%, respectively. The population characteristics of the subjects stratified by sex are shown in Table 1. In both studies, women were found to be younger than men and have lower WC, lower DBP, higher SBP, higher serum TC, higher serum HDL-c, and higher serum TG. Women were less likely to be drinker, current smoker, and to spend less time on exercise than men.

**Table 1:** Characteristics of study participants of both cross-sectional and longitudinal gene associated studies among a Chinese Aged Population Sample.

	Cross-sectional Study (2 014)			Longitudinal Study (742)		
Variables	Men	Women	*p*	Men	Women	*p*
**N**	798	1,216		279	463	
**Age (years)**	72.37±6.91	70.35±6.20	<0.001*	77.17±4.63	75.02±4.72	<0.001*
**BMI (kg/m^2^)**	25.00±3.03	25.03±3.63	0.848	25.26±2.87	25.69±3.31	0.061
**Waist circumference (cm)**	91.34±8.40	84.34±8.91	<0.001*	89.50±8.31	85.40±8.20	<0.001*
**Smoke, N (%)**			<0.001*			<0.001*
Never smoker,	369 (46.2)	1104 (90.8)		131 (47.0)	410 (88.6)	
Former smoker	253 (31.7)	61 (5.0)		81 (29.0)	19 (4.1)	
Current smoker	176 (22.1)	51 (4.2)		67 (24.0)	34 (7.3)	
**Current drinker, N (%)**	377 (47.2)	100 (8.2)	<0.001*	80 (28.7)	29 (6.3)	<0.001*
**Education level, N (%)**			<0.001*			<0.001*
Primary school or lower	144 (18.0)	420 (34.5)		68 (24.4)	222 (47.9)	
Middle school or High school	279 (35.0)	536 (44.1)		107 (38.4)	170 (36.7)	
University or above	375 (47.0)	250 (21.4)		104 (37.3)	71 (15.3)	
**Physical activity, N (%)**			0.122			<0.001
< one hour per day	99 (12.4)	184 (15.1)		25 (9.0)	86 (18.6)	
1 - 3 hours per day	629 (78.8)	911 (74.9)		220 (78.9)	350 (75.8)	
≥ 4 hours per day	70 (8.8)	121 (10.0)		34 (12.2)	26 (5.6)	
**Family diseases history, N (%)**						
Coronary heart disease	181 (22.7)	270 (22.2)	0.801	52 (18.8)	76 (16.4)	0.412
Diabetes	126 (15.8)	237 (19.5)	0.035*	20 (7.2)	39 (8.4)	0.559
Hypertension	282 (35.3)	563 (46.3)	<0.001*	91 (32.9)	192 (41.5)	0.020*
**SBP, mmHg**	136.72±17.8	140.60±20.8	<0.001*	133.6±17.9	135.57±21.6	0.182
**DBP, mmHg**	78.40±9.6	76.72±9.9	<0.001*	78.47±9.6	77.84±11.1	0.430
**FPG, mmol/L**	6.12±1.5	6.10±1.7	0.771	5.89±1.5	6.01±1.7	0.324
**Serum HDL-c, mmol/L**	1.31±0.3	1.47±0.3	<0.001*	1.26±0.3	1.41±0.3	<0.001*
**Serum TG, mmol/L**	1.52±0.8	1.78±0.9	<0.001*	1.39±0.9	1.71±1.2	<0.001*
**Serum TC, mmol/L**	4.95±0.9	5.50±1.0	<0.001*	4.97±0.9	5.48±5.0	<0.001*
**CHD, N (%)**	204 (25.6)	270 (22.2)	0.188	90 (33.7)	199 (42.4)	0.020*
**Stroke, N (%)**	111 (13.9)	144 (11.8)	0.172	29 (10.9)	67 (14.3)	0.181

The results are presented as mean ± SD or N (%).

*statistically significant.

doi:10.1371/journal.pone.0100548.t001

Of the 23 SNPs, SNP rs328 in the gene *LPL* was found to be departed from Hardy-Weinberg Equilibrium in the control group (data not shown) and was excluded from all analyses. Strong LD was observed between rs8050136 and rs9939609 in *FTO* gene (r^2^ = 0.99), between rs4506565 and rs7903146 in *TCF7L2* gene (r^2^ = 0.98), and between rs4712523 and rs7754840 in *CDKAL1* gene (r^2^ = 0.94). However, LD was low between rs1387153 and rs10830963 in *MTNR1B* gene (r^2^ = 0.67), and between rs1801131 and rs1801133 in *MTHFR* gene (r^2^ = 0.18).

### Associations Of Snps With Metabolic-Related Traits

We used multivariable logistic regression models to examine the associations of candidate SNPs with MetS and metabolic-related traits, adjusted for age, sex, BMI, and lifestyle factors (leisure time physical activity, alcohol consumption, smoking status, and educational level). In cross-sectional analyses, genotype TT of rs4402960 located within gene *IGF2BP2* was associated with overweight (OR = 0.479, 95% CI: 0.316–0.724, *p* = 0.001) and genotype CA of rs1801131 in gene *MTHFR* was associated with hypertension (OR = 1.560, 95% CI: 1.194–2.240, *p* = 0.001) after adjusting for multiple testing. However, these statistically significant associations were not observed in the longitudinal analyses (Table 2).

**Table 2:** Results from Cross-sectional and Longitudinal Analyses of the Associations of Selected SNPs with Obesity and Other Metabolic-related Traits among a Chinese Aged Population Sample.

Phenotype	Gene	SNP	Genotype	Cross-sectional Study				Longitudinal Study			
				N = 2 014				N = 742			
				Case	Control	OR (95%CI)	*p*	Case	Control	OR(95%CI)	*p*
Overweight 1	*ACE*	rs4343	AA#/AG	383/458	456/480	1.136 (0.934,1.382)	0.202	117/159	122/139	1.130 (0.797,1.602)	0.492
			AA#/GG	383/132	456/180	1.453 (1.072,1.964)	0.016	117/44	122/21	1.966 (1.108,3.592)	0.021
	*ADIPOQ*	rs1501299	CC#/AC	73/349	72/434	0.773 (0.637,0.939)	0.009	177/119	140/114	0,810 (0.571,1.149)	0.238
			CC#/ AA	73/551	72/535	1.011 (0.703,1.455)	0.952	177/24	140/28	0.632 (0.345,1.157)	0.137
	*KCNQ1*	rs2237892	CC#/CT	433/438	480/487	1.007 (0.831,1.220)	0.926	132/151	137/130	1.220 (0.864,1.723)	0.258
			CC#/TT	433/102	480/74	1.630 (1.154,2.301)	0.006	132/37	137/15	2.520 (1.297,4.896)	0.006
	*IGF2BP2*	rs4402960	GG#/GT	594/339	575/389	0.878 (0.724,1.065)	0.187	13/103	25/106	0.689 (0.485,0.979)	0.060
			GG#/TT	594/40	575/77	0.479 (0.316,0.724)	0.001*	13/204	25/151	0.382 (0.187,0.779)	0.008
	*CDKN2A/B*	rs10811661	TT#/CT	311/452	293/536	0.790 (0.639,0.978)	0.030	105/147	82/132	0.915 (0.623,1.345)	0.652
			TT#/CC	311/210	293/212	0.916 (0.706,1.188)	0.508	105/68	82/68	0.761 (0.482,1.201)	0.241
T2DM^2^	*KCNQ1*	rs2237892	CC#/CT	321/288	592/637	0.819 (0.666,1.007)	0.059	65/46	183/225	0.552 (0.353,0.862)	0.009
			CC#/TT	321/43	592/133	0.577 (0.390,0.854)	0.006	65/14	183/47	0.817 (0.409,1.633)	0.568
Dyslipidemia^2^	*INSIG*	rs7566605	GG#/CG	633/717	230/197	1.335 (1.077,1.704)	0.009	186/217	42/42	1.108 (0.660,1.859)	0.699
			GG#/CC	633/174	230/63	0.969 (0.690,1.361)	0.763	186/59	42/12	0.934 (0.440,1.983)	0.859
Hypertension^2^	*MTHFR*	rs1801131	AA#/CA	999/350	488/134	1.560 (1.194,2.240)	0.001*	228/69	131/29	1.191 (0.693,2.046)	0.528
			AA#/CC	999/26	488/17	0.876 (0.418,1.833)	0.725	228/2	131/4	0.202 (0.030,1.360)	0.100
		rs1801133	CC#/CT	284/620	140/322	1.052 (0.795,1.393)	0.722	55/122	26/94	0.625 (0.344,1.134)	0.122
			CC#/TT	284/471	140/177	1.533 (1.108,2.121)	0.010	55/122	26/64	1.241 (0.654,2.355)	0.509
	*CDKAL1*	rs4712523	AA#/AG	443/705	200/309	1.621 (0.544,4.836)	0.386	104/155	57/73	1.066 (0.666,1.707)	0.790
			AA#/GG	443/227	200/130	10.911 (1.634,72.851)	0.014	104/40	57/34	0.576 (0.308,1.077)	0.084
		rs7754840	GG#/CG	462/702	205/306	0.583 (0.197,1.727)	0.330	107/155	59/71	1.077 (0.674,1.720)	0.757
			GG#/CC	462/211	205/128	0.069 (0.010,0.465)	0.006	107/37	59/34	0.541 (0.288,1.017)	0.056

1 : Adjusted for sex, age, life style factors (exercise, alcohol consumption, smoking status, and educational level).

2 : Model 1 + BMI.

# : Reference genotype.

*: Statistically significant

doi:10.1371/journal.pone.0100548.t002

The associations of SNP rs4402960 and SNP rs2237892 with overweight were similar in our study and were both identified to be associated with diabetes before. That suggested a potential gene-gene interaction of gene *IGF2BP2* with *KCNQ1* on overweight in our study. So we examined this interaction using a logistic regression model, however the interaction was not statistically significant (data not shown).

### Associations Of Snps With Mets

We found no statistically significant association of SNPs with MetS in both analyses.

## Discussion

The associations of the variations of these candidate SNPs with obesity and various traits of the MetS have been examined in many populations. However, to our knowledge, this is the first study to investigate so many SNPs among a large elderly Chinese population. Elderly subjects are expected to have higher cumulative exposure to environmental risk factors and thus be more likely to be affected by gene-environment interaction than younger people. It is unknown whether the environmental influences would dilute the contribution of susceptible genotypes to metabolic-related traits or, rather, would make the phenotypes conferred by the susceptible genotypes more likely to show up [27]. Therefore, it is meaningful to investigate these genetic associations among aged participants.

We carried out two observational studies among Chinese elders to explore associations of a total of 22 SNPs located within 16 genes with MetS and metabolic-related traits, including overweight and obesity, T2DM, hypertension and dyslipidemia. In the cross-sectional analyses, statistically significant associations of the SNP rs4402960 located in gene *IGF2BP2* with overweight and the SNP rs1801131 in gene *MTHFR* with hypertension were observed in our model 1 adjusting for age, sex, life style factors (leisure time physical activity, alcohol consumption, smoking status, and educational level). Further inclusion of BMI in the model 2 did not change the statistical significance and the magnitude of the association, indicating that the association may be independent of BMI. The association of rs1801131 with hypertension was not statistically significant in the longitudinal analyses, but this is possibly because of the low statistical power due to a small sample size.

**Figure 1:**
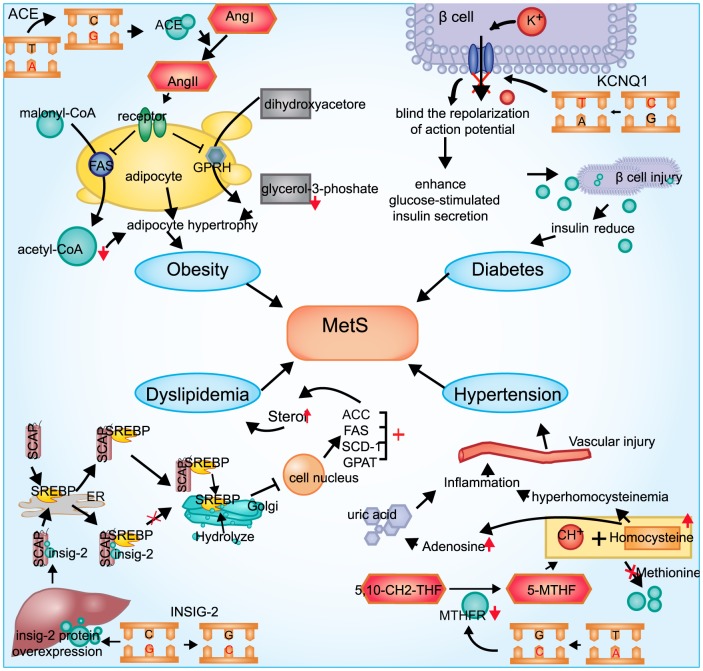
Functional associations between the SNPs and the phenotypes. The figure depicted the biological functional associations between four SNPs and different traits of MetS. *KCNQ1* (potassium voltage-gated channel KQT-like subfamily, member 1) is a gene encoding the poreforming subunit of a voltage-gated K+ channel (KvLQT1) that plays a key role for the repolarization of the cardiac action potential as well as water and salt transport in the beta cells. T allele variant might inhibit the KV-channels in beta cells and enhance glucose-stimulated insulin secretion, which leads to an increased risk of diabetes. *ACE* gene encoding the angiotensin (Ang) and transform Ang I into Ang II, and the activation of Ang IImight increase the storage of TG by influencing the glycolysis process and lead to the adipocyte hypertrophy. C allele variant of the *INSIG2* gene was involved in the reversed cholesterol transport by an interaction with sterol regulatory element-binding proteins (SREBPs), which are transcription factors that activate the synthesis of cholesterol and fatty acids in the liver and other organs. In addition, A to C transition at nucleotide 1298 (A1298C, rs1801131) of the coding sequence in gene *MTHFR*, have been shown to be the most frequent genetic causes for mild hyperhomocysteinemia, and a high plasma concentration of homocysteine may predispose to atherosclerosis by injuring the vascular endothelium, which might result in hypertension.

We also depicted the functional associations between some SNPs and phenotypes (Fig.1). IGF2 is a polypeptide growth factor that plays an important role in growth and development, and it stimulates insulin action. *IGF2BP1* binds to the leading 3 mRNA in the 5′-UTR of the *IGF2* gene to regulate *IGF2* translation [28]. However, the biological function of *IGF2BP2* is not well-studied. Previous studies have shown that the T allele of the SNP rs4402960 was a risk factor for diabetes [29]. In the present study, we found no statistically significant association between rs4402960 and diabetes. However, variant allele T was negatively associated with overweight. To our knowledge, this is the first study that reports such a protective association of this SNP with overweight. In the study by Xia Li *et al*, rs11705701, a SNP in strong LD with rs4402960, was identified to be associated with percent body fat in Mexican Americans [30]. In addition, SNP rs2237892 located within gene *KCNQ1* was found to be associated with both overweight and obesity and diabetes at first [19,[31],[32]. Variant allele T of rs2237892 was associated with a higher prevalence of overwight, but was with a lower prevalence of diabetes in our study, which was consistent with previous studies [31],[33]–[35]. Biologically, *KCNQ1* is a gene encoding the poreforming subunit of a voltage-gated K+ channel that plays a key role in the repolarization of the cardiac action potential as well as water and salt transportation in the beta cells [19],[32],[36]. KV-channel knock-out in rat islets as well as pharmacological inhibition of KV-channels in rat beta cells have been reported to enhance glucose-stimulated insulin secretion [37]–[39]. Meanwhile, *IGF2BP2* were also associated with reductions in first-phase insulin secretion in some studies [40],[41]. Moreover, both SNPs were located at 11p15 of chromosome, variants of which were confirmed to be associated with the Beckwith-Wiedemann syndrome (BWS). The associations mentioned above suggested a possible interaction between rs4402960 and rs2237892 for overweight and obesity in our study. We tested this interaction but found no statistical significance. Further studies of these two SNPs are necessary.

*INSIG1* and *INSIG2* play important roles in regulating cholesterol or TG synthesis, mainly in the liver. rs7566605, which is located 10 kb upstream of *INSIG2*, was reported to have the strongest association with obesity among 86 604 SNPs [42]. Biological functional evidence depicted the *INSIG2* gene from the very start as a candidate gene for obesity as being involved in the reversed cholesterol transport by an interaction with sterol regulatory element-binding proteins (SREBPs) [43], which are transcription factors that activate the synthesis of cholesterol and fatty acids in the liver and other organs [44]. Even so, studies on the SNP-obesity associations reported inconsistent results [45]–[52]. In the present study, we did not obtain statistically significant association between SNP rs7566605 and overweight and obesity. The relationship between rs7566605 with dyslipidemia was also not statistically significant after correcting for multiple testing. A Japanese population study reported that rs7566605 was associated with dyslipidemia in heterogeneous co-dominant genetic model, which known as the first report to identify this associations [53]. So we analyzed the relationship between SNP rs7566605 and dyslipidemia in different heterogeneous models, adjusting for multiple covariates. We found subjects with the CG heterozygote had 1.368-fold (95% CI: 1.107–1.192) increased in OR for dyslipidemia compared to the combination of genotype GG and genotype CC (data not shown). This result also suggested a heterogeneous co-dominant genetic model in our study.

*MTHFR* gene showed a strong association with hypertension in our cross-sectional analyses, and OR was similar to the result of a previous meta-analysis among Asian population [54]. A common C to T transition at nucleotide 677 (C677T, rs1801133) and A to C transition at nucleotide 1298 (A1298C, rs1801131) of the gene coding sequence, have been shown to be the most frequent genetic causes for mild hyperhomocysteinemia [55],[56]. High plasma concentration of homocysteine may predispose individuals to atherosclerosis by injuring the vascular endothelium, which might result in hypertension.

In the present study, the results suggested that these candidate SNPs were significant genetic contributors to the phenotypes among elderly, indicating that the associations of these SNPs with MetS and metabolic-related traits observed in young population are also able to be observed in elders. Advantages and limitations of our study should be taken into account when interpreting the findings. Our study has the advantage that subjects without phenotypes were from old population, so the probability that they will develop metabolic disorders in the future might be relatively low. Therefore, the metabolic-related traits in older subjects may faithfully reflect their genetic makeup. This hypothesis was also mentioned in a study before [27]. However, our study also has several limitations that subjects carrying certain genotypes combined with the metabolic-related traits may not survive to the old age, which may lead to biased conclusions of the associations between these genotypes and the metabolic-related traits among elderly. Participants were enrolled from communities or streets in Wanshoulu area, which is a commodity-type residential quarter where residents are usually with different occupations and different backgrounds. The potential genetic bias might be exist but is expected to be low. However, we could not examine the extent of this bias. We conducted a longitudinal analysis to further investigate the results from cross-sectional study, but the small sample size limited our statistical power to detect these associations. Since the metabolic syndrome is highly complex and polygenic, a large sample size would be needed.

## Conclusions

In conclusion, among an elderly Chinese population, we found statistically significant associations between SNP rs4402960 with overweight and SNP rs1801131 with hypertension in cross-sectional analyses. No statistically significant association was found in longitudinal analyses, possibly due to the limited statistically power. No SNP was found to be associated with MetS in our analyses with statistically significance. Our findings suggest that some of the SNPs identified among younger people are also replicated in elderly. However, due to the limitations of our study, further investigations on these associations are guaranteed.
